# Immediate Biomechanical Effects of Manual and Tool-Assisted Myofascial Release on the Erector Spinae Muscle

**DOI:** 10.3390/s25227021

**Published:** 2025-11-17

**Authors:** Yueh-Ling Hsieh, Heng-Yi Lin, Andy Chien

**Affiliations:** 1 Department of Physical Therapy, China Medical University, Taichung 406040, Taiwan; sherrie@mail.cmu.edu.tw (Y.-L.H.); u114030017@cmu.edu.tw (H.-Y.L.); 2 Department of Biomedical Engineering, College of Medicine and College of Engineering, National Taiwan University, Taipei 106319, Taiwan

**Keywords:** erector spinae muscles, myofascial release, elasticity, tone, stiffness, MyotonPRO

## Abstract

The biomechanical characteristics of the erector spinae muscles are crucial for evaluating treatment effectiveness. Although it is widely believed that myofascial release directly impacts muscle biomechanics, there has been limited research directly comparing manual (MMR) and tool-assisted (TMR) applications. This study aimed to fill this gap by investigating the immediate biomechanical effects of MMR and TMR on the erector spinae muscles, using the MyotonPRO device to measure and compare changes in muscle tone, stiffness, and elasticity. Thirty healthy adult physical therapy students (21.19 ± 1.93 years) were recruited and randomly assigned to either the MMR or TMR group. Biomechanical properties (elasticity, tone, and stiffness) were measured before and immediately after three sets of 15 repetitions of the assigned intervention. Post-intervention, the MMR group showed a significant decrease in muscle stiffness and tone (*p* < 0.0125), while the TMR group showed no significant changes in any of the measured parameters (all *p* > 0.05). A comparison of the percentage change from baseline also revealed significant differences in elasticity, stiffness, and tone between the two groups (*p* < 0.0125). This study demonstrates that MMR produces a significant and immediate reduction in erector spinae muscle stiffness and tone, an effect not observed with TMR.

## 1. Introduction

The erector spinae muscles, as key stabilizers of the spine, can undergo biomechanical changes that lead to abnormal body movements and may cause low back pain [1,2]. Abnormally elevated muscle tension can lead to increased stiffness, which in turn impairs blood and lymphatic circulation, slowing down tissue metabolism [3]. This process can result in a buildup of lactic acid, causing muscle tightness and contraction and ultimately altering the muscle’s biomechanical properties [4]. Consequently, these muscle biomechanical characteristics serve as crucial objective indicators of tissue health [5,6,7,8], essential for erector spinae muscles in terms of understanding the etiology of chronic low back pain and for evaluating treatment efficacy.

Myofascial release is a technique that may exert the most direct influence on the biomechanical properties of muscle. It involves the use of direct manual or tool-assisted methods to massage and stretch stiff areas, aiming to alleviate internal myofascial tension [9,10]. Manual myofascial release technique (MMR) is a traditional approach where a clinician uses their hands, fingers, and elbows to apply sustained force to the muscle and fascia, targeting adhesions and trigger points, which is widely used clinically to treat chronic low back pain [11,12,13]. While various myofascial release techniques continue to evolve, recent literature has increasingly focused on self-myofascial release using tool-assisted myofascial release (TMR) techniques [14,15,16,17]. These techniques are performed by individuals themselves using tools such as foam rollers, massage balls, and other devices to achieve the purpose of self-myofascial release [10]. TMR has gained popularity because it adheres to the same principles as MMR but allows for more regular and frequent application without the need for a therapist’s intervention [18]. Despite the growing support for the clinical application of TMR, there is a notable lack of direct assessment regarding the effects of changes in muscle biomechanical properties between MMR and TMR techniques.

Myotonometry is a non-invasive technique used to quantitatively assess muscle tone, stiffness, and elasticity. These specific measurements are referred to as myometric parameters. MyotonPRO, a handheld, non-invasive myotonometer [19], employs complex algorithms to numerically determine muscle tone, stiffness, and elasticity in relatively superficial muscles, based on the principle that skeletal muscle stiffness reflects its resistance to deformation [20]. These myometric parameters offer a precise and objective means of evaluating a muscle’s broader biomechanical properties and its mechanical behavior. The reliability and informational value of Myoton technology have been confirmed [21,22,23].

The use of MMR and TMR has been shown to induce changes in muscle biomechanical properties. However, there is still debate regarding the time course of these changes. The literature suggests that alterations in muscle stiffness and tone may appear with a delay, rather than immediately [24]. Therefore, understanding the acute response is crucial for grasping the underlying mechanisms and providing immediate guidance for subsequent treatment. Accordingly, this study aimed to investigate the immediate biomechanical effects of MMR and TMR myofascial release on the erector spinae muscles and to compare the efficacy of these two techniques by using the MyotonPRO device to detect changes in muscle tone, stiffness, and elasticity. It was hypothesized that both MMR and TMR would lead to a significant, immediate effects in biomechanical properties and that MMR would be more effective than TMR. Ultimately, the results were expected to enhance the fundamental understanding of how these interventions influence the biomechanics of paraspinal muscles.

## 2. Materials and Methods

### 2.1. Participants

A total of 30 healthy adult volunteers (14 males, 16 females) aged between 20 and 25, all of whom were physical therapy students familiar with myofascial release techniques, were recruited for this study. Inclusion criteria required participants to have palpable taut bands and tenderness in their lower back muscles, without a history of major pain complaints or spinal, hip, or pelvic diseases. Exclusion criteria included individuals with symptoms of radiculopathy, trauma-induced lower back pain, diagnosed spinal deformities (e.g., scoliosis or spondylolisthesis), lumbar disc herniation, spinal infections or tumors, rheumatic diseases, a history of spinal surgery, or a treated history of hip or pelvic disorders. Furthermore, individuals with a body mass index (BMI) of 30 kg/m^2^ or higher (as defined by MyotonPRO guidelines) were also excluded. All participants provided written informed consent prior to their involvement. The study protocol adhered to the ethical principles of the Declaration of Helsinki and was approved by the Institutional Review Board of China Medical University Hospital (No.: CMUH110-REC2-071, 10 May 2021).

### 2.2. Experimental Design

This study adopted a randomized trial design to compare the immediate effects of MMR and TMR on the biomechanical properties of the spinal erector muscles. Before any intervention, baseline measurements of muscle tone, stiffness, and elasticity were collected using the MyotonPRO device in a prone position at the third (L3), fourth (L4), and fifth (L5) lumbar levels of the spinal erector muscles. Following baseline testing, participants were randomly assigned via a computer-generated number sequence to either the MMR (*n* = 5 males, 10 females) or TMR (*n* = 6 males, 9 females) intervention group. Subsequently, participants received their assigned myofascial release intervention. Immediately after the intervention, post-intervention MyotonPRO measurements were collected from the same pre-marked L3, L4, and L5 levels of the spinal erector muscles in the prone position.

### 2.3. Myometric Parameter Measurement

The myometric parameters of the erector spinae muscle—specifically stiffness and elasticity—were measured using a MyotonPRO device (Myoton Ltd., Tallinn, Estonia). To ensure consistent technique and procedure, all measurements were performed by a single, experienced researcher who was professionally trained on the MyotonPRO device. The re-test reliability of the instrument was also assessed before the main study to confirm that the data produced was reliable and stable. This device applies a mechanical impulse to the muscle and uses a built-in accelerometer to record the subsequent oscillations. These oscillations are then used to calculate the myometric parameters, which serve as objective indicators of the muscle’s biomechanical properties: natural oscillation frequency (Hz), characterizing tone or tension, stiffness (N/m), a measure of the muscle’s resistance to deformation, and elasticity (logarithmic decrement), an indicator of the muscle’s viscosity or ability to dissipate energy during oscillation.

**Figure 1:**
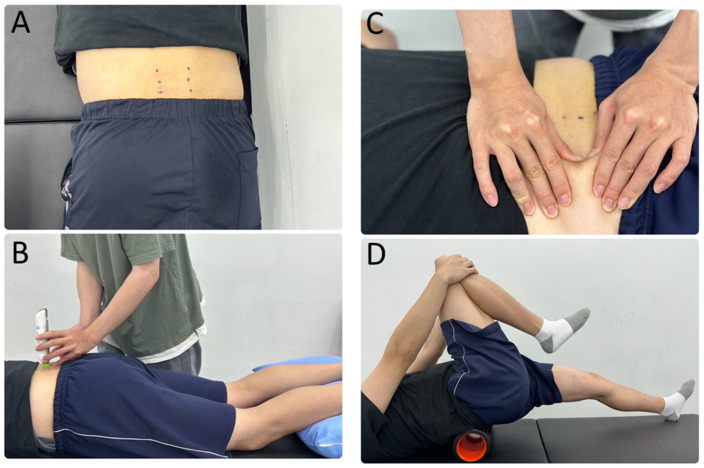
MyotonPRO evaluation and myofascial release techniques: (**A**) shows the palpation and marked points for erector spinae muscles L3, L4, and L5 levels, (**B**) shows the MyotonPRO testing position, (**C**) demonstrates the manual myofascial release (MMR) technique and position, and (**D**) illustrate tool-assisted myofascial release (TMR) using a foam roller.

These anatomical locations were precisely marked in the prone position to ensure consistency and fixation of the measurement points throughout this study (Figure 1A). Before any intervention, participants were positioned in a relaxed prone position (Figure 1B). The bilateral testing points for the spinal erector muscles were carefully located approximately 3 cm lateral to spinous processes of the L3, L4, and L5 levels. The probe was placed vertically and stably on the skin surface covering the bilateral lumbar spinal erector muscle bellies at the L3, L4, and L5 levels, yielding data from six points. Measurement began at L3 level of the spinal erector muscles. Measurements were then taken sequentially from L3 to L5, and this order was repeated on the contralateral side of the body. Each individual level measurement took about 5 s to complete. The entire process for all six spinal erector muscle sites was completed in approximately 30 to 40 s. A controlled preload of 0.18 N was first applied to initially compress the subcutaneous tissue, followed by a 15-millisecond mechanical impulse of 0.4 N, which elicited a damped natural oscillation within the muscle. The device automatically displayed these data, recording the natural oscillation frequency (Hz; representing muscle tone), dynamic stiffness (N/m), and elasticity (logarithmic decrement, characterizing viscoelasticity). Prior to statistical analysis, it was confirmed that there were no significant differences among the data from the six measurement positions (L3, L4, and L5 showed no difference, nor did the left and right sides). For each participant, the raw values for each myometric parameter (tone, stiffness, and elasticity) were averaged across all six measurement locations to yield single pre- and post-intervention mean values. The percentage change was then calculated as: (post-intervention value − pre-intervention value)/pre-intervention value × 100%.

### 2.4. Myofascial Release Intervention Procedures

For the MMR group, a single, experienced therapist was trained in a standardized protocol to apply consistent manual force, targeting a range of 8.9–26.7 N (2.0–6.0 lb). This target range was established during pilot testing using a microFET^®^2 Digital Handheld Dynamometer Algometer (Hoggan, Salt Lake City, UT, USA). Although the device was not used during the actual treatment, the therapist was trained to maintain the force range based on consistent perceived exertion, which was validated before this study. For the intervention, participants were in a prone position and received a massage from the therapist. The therapist was instructed to apply a consistent, sustained force of 2.0–6.0 lb for 30 s along the taut bands of the erector spinae muscles (specifically the L3–L5 region) to ensure a uniform treatment dose. The intervention consisted of three sets of 15 repetitions, with a 1 min rest between sets (Figure 1C).

For the TMR group, participants, all of whom were physical therapy students, completed a standardized training session before randomization. This was carried out to ensure all participants performed the procedures with an identical protocol, speed, and force, minimizing individual differences. For the self-myofascial release, participants used a foam roller (GRID 1.0, TriggerPoint, Durham, NC, USA), positioning the L3–L5 region of their lower back on the device. The roller is a 33 cm long, 14 cm diameter device constructed with a durable EVA foam surface over a hollow core. To maintain spinal stability and muscle relaxation, all participants were instructed to randomly flex one of their legs, and they maintained this position consistently throughout the experiment; the legs were not alternated within or between sets. A single repetition was defined as one full rolling cycle (a single forward and backward motion) on the foam roller. Participants then performed rolling motions following a protocol identical to the MMR group: three sets of 15 repetitions with a 1 min rest between sets. Each repetition of the foam rolling lasted 30 s (Figure 1D). To ensure consistent speed and force, participants were guided by a metronome and instructed to apply approximately 50% of their body weight onto the foam roller. Researchers provided continuous verbal reminders and monitored the entire process to ensure strict adherence to the protocol. Any notable deviations were recorded to enhance data validity and reliability. All participants were required to commit to strictly adhering to the assigned intervention protocol without self-adjustment.

Upon completion of the intervention (MMR or TMR), post-intervention measurements were initiated immediately. It took approximately 15 to 20 s from the end of the intervention to the start of the first muscle site measurement.

### 2.5. Statistical Analysis

Descriptive statistics, including means and standard deviations (mean ± SD), were calculated for all demographic and myometric parameters. The normality of data distribution was confirmed using the Shapiro–Wilk test (*p* > 0.05). A two-way mixed-model ANOVA was performed to examine the effects of the between-subjects factors (Gender and Group) and the within-subjects factor (Time, representing the duration from pre- to post-intervention measurements) on elasticity, stiffness, and tone.

To investigate changes in pre- and post-intervention measurements within each group, post hoc paired *t*-tests were conducted. Furthermore, when significant group differences were observed, independent *t*-tests were used as a post hoc analysis to compare the intervention groups. Eta-squared (η^2^) effect sizes were calculated for all ANOVA results. This metric quantifies the proportion of variance in the dependent variable explained by an independent variable or factor, providing a measure of the effect’s practical significance. To control for Type I error inflation due to multiple comparisons, a Bonferroni correction was applied, setting the adjusted level of significance to α = 0.0125. The level of significance for all other statistical tests was set at α = 0.05. All statistical analyses were conducted using SPSS software (version 22.0, IBM Corp., Armonk, NY, USA).

## 3. Results

Based on Table 1, the MMR and TMR groups were comparable in their demographic and physical characteristics. There were no significant differences in age, weight, height, or BMI between the two groups for either male or female participants (*p* > 0.05 for all). The results detailing the between- and within-subject effects on muscle elasticity, stiffness, and tone were analyzed using a mixed-model ANOVA and are presented in Table 2. For elasticity, a significant interaction was found between Time and Group (F(1, 26) = 7.023, *p* = 0.014, η^2^ = 0.213, power = 0.723), with no other main effects or interactions being statistically significant (*p* > 0.05). For stiffness, there was a significant main effect of Time (F(1, 26) = 18.772, *p* < 0.001, η^2^ = 0.419, power = 0.986), a significant interaction between Time and Group (F(1, 26) = 10.107, *p* = 0.004, η^2^ = 0.280, power = 0.864), and a significant main effect of Gender (F(1, 26) = 5.503, *p* = 0.027, η^2^ = 0.175, power = 0.617). Regarding tone, a significant interaction was observed between Time and Group (F(1, 26) = 12.643, *p* = 0.001, η^2^ = 0.327, power = 0.928), and a significant main effect of Gender was also found (F(1, 26) = 4.329, *p* = 0.047, η^2^ = 0.143, power = 0.517).

**Figure 2:**
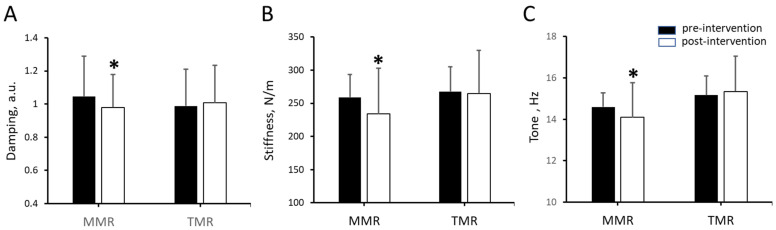
Immediate effects on myometric parameters after intervention in MMR and TMR groups. The mean ± standard deviation for (**A**) elasticity, (**B**) stiffness, and (**C**) tone were measured at pre- and post-intervention time points for MMR and TMR groups. * indicates a significant difference between the pre- and post-intervention values within a group (*p* < 0.0125).

**Figure 3:**
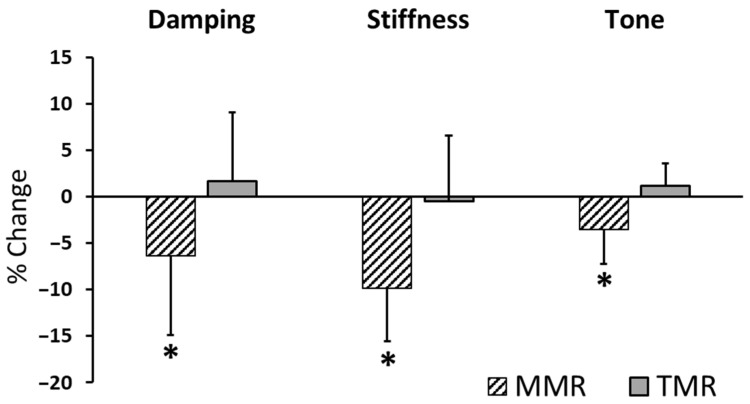
Mean percentage change (with standard deviation) in muscle elasticity, stiffness, and tone after MMR and TMR. The percentage change was calculated as (post-treatment value − pre-treatment value)/pre-treatment value × 100%. An asterisk (*) indicates a statistically significant difference from the baseline, with a *p*-value less than 0.0125.

Table 3 showed that paired-samples *t*-tests were used to investigate the immediate effects of each treatment by comparing myometric parameters before and after the intervention. Post-intervention, the MMR group experienced significant decreases in stiffness and tone (*p* < 0.0125 for both), though the decrease in elasticity (*p* = 0.016) was not statistically significant. Following the intervention, the TMR group showed no significant changes in elasticity (*p* = 0.344), stiffness (*p* = 0.525), or tone (*p* = 0.088) (Figure 2). A comparison of the percentage changes revealed significant differences in elasticity, stiffness, and tone between the MMR and TMR groups (*p* < 0.0125). For the MMR group, all three measured properties—elasticity, stiffness, and tone—showed negative percentage changes, signifying a reduction in their values after the intervention (Figure 3).

## 4. Discussion

This study aimed to investigate the immediate biomechanical effects of MMR and TMR on the erector spinae muscle, as measured by the MyotonPRO device. Our results show a significant immediate decrease in stiffness and tone of erector spinae muscles in the MMR group after the intervention, whereas the TMR group demonstrated no significant difference. The substantial effect sizes for these changes, specifically the large η^2^ values (>0.14) for the MMR intervention, suggest the observed changes in elasticity, stiffness, and tone are of a clinically meaningful magnitude, not just statistically significant. These findings offer new insights into the quantitative changes in muscle tone, stiffness, and elasticity following these interventions, suggesting a possible mechanism by which fascial release techniques influence erector spinae muscle characteristics. The MyotonPRO, a device previously validated for its reliability in quantifying muscle properties [8,25,26], successfully detected these changes. The significant main effect of Time on the Myoton stiffness and tone parameters further supports the effectiveness of the MMR intervention in altering biomechanical properties of erector spinae muscle. This quantitative evidence, derived from a non-invasive tool, provides a solid foundation for the clinical effects of techniques performed by therapists.

When assessing muscle biomechanical properties (e.g., tone, stiffness, and elasticity), ultrasound and myotonometry are two widely used non-invasive tools. While ultrasound provides highly accurate, image-guided measurements, making it particularly suitable for precise localization, it is highly operator-dependent and the equipment is expensive. In contrast, myotonometry offers an objective quantification of these properties by applying a mechanical impulse and analyzing the oscillation frequency and its decay in the muscle tissue. The MyotonPRO’s reliability and informational value have been validated [21], and its correlations with other methods, such as electromyography (EMG) and shear wave ultrasound elastography, have been observed [27,28]. Numerous studies have also found the MyotonPRO to be capable of measuring the lumbar muscles and fascia in healthy individuals, demonstrating moderate to very high reliability in assessing muscle stiffness [28,29]. Furthermore, its validity is empirically supported, as myotonometer-measured stiffness has been shown to correlate significantly with the rate of force development during isometric contractions [30]. Compared to other assessment tools like ultrasound, myotonometry offers distinct advantages of ease of use and cost-effectiveness, as well as the ability to measure multiple parameters simultaneously. While the myotonometer lacks imaging capabilities and has a limited assessment depth, its superior reliability and validity make it an effective and reliable tool for screening the biomechanical properties of superficial muscles.

Some studies using ultrasound and force measurement instruments have found that the stiffness index of the posterior thoracolumbar fascia (calculated from the slope of the loading curve) in healthy men significantly and immediately decreases after MMR [31]. Similarly, research employing B-mode ultrasound, real-time elastography, and a durometer has shown that MMR can simultaneously improve deep fascial gliding and muscle stiffness [32]. In the treatment of primary dysmenorrhea, MMR is effective and significantly reduces lower abdominal muscle tone. Compared to transcutaneous electrical nerve stimulation and exercise training, this therapy shows better results in reducing muscle tone, and its efficacy is sustained after the treatment ends [33]. Research results indicate that MMR techniques can effectively reduce the stiffness of the thoracolumbar fascia and erector spinae muscles in patients with low back pain, while also decreasing pain intensity [34]. Our results, showing a significant immediate decrease in muscle stiffness and tone in the MMR group after the intervention, are consistent with the findings of much of the existing literature. The precise mechanisms of MMR remain unclear, but they likely involve a combined effect of biomechanical, neurophysiological, and psychological factors. Biomechanically, manual force may directly alter fascial tissue by breaking down collagen adhesions and re-aligning soft tissue, thereby improving its viscosity, elasticity, and mobility [35]. Neurophysiologically, these techniques can reduce muscle tone and pain by stimulating mechanoreceptors and proprioceptors, which initiates a cascade of effects within the central and autonomic nervous systems, activating descending inhibitory pain pathways [36]. The psychological component, including the therapeutic touch and a positive therapist-patient relationship, can also induce a relaxation response that indirectly influences muscle tone [37].

In addition, our study found that the TMR group showed no statistically significant changes in elasticity, stiffness, or tone after the intervention. This finding is particularly noteworthy as it challenges the common assumption that MMR and TMR, being based on similar principles, should produce similar immediate biomechanical effects. Previous research has found that after four 45-s foam rolling sessions (i.e., TMR), the compression stiffness of the anterior thigh decreased by 15% to 24%, with these reductions being more significant 10 min post-intervention than immediately after [24]. Another study using shear wave elastography to evaluate the viscoelastic behavior of the muscle-fascia complex observed a small immediate decrease in the elastic modulus after rolling (effect size: 0.21) [38]. Furthermore, a study using acoustic radiation force imaging to assess mechanical tissue properties of the thigh showed that in participants with prior foam rolling experience, the stiffness of the iliotibial band significantly decreased after 30 min (−13%), while the muscle tissue itself remained unaffected [39]. Research using a durometer and ultrasound image analysis to evaluate tissue changes in 18 sedentary men after a 3 min foam rolling massage on the right thigh found that foam rolling significantly reduced fascial stiffness and improved the proximal gliding ability of the fascia lata during passive knee extension [40]. A study showed that short-duration, high-intensity self-induced myofascial release (i.e., TMR) performed after a warm-up did not produce a significant and reliable immediate improvement in jump performance [41]. Thus, despite having no negative effects, TMR may not directly produce an immediate effect.

In light of recent literature, including Koruk and Rajagopal’s comprehensive review on viscoelastic parameters used for engineering and soft materials [42], the use of the term “elasticity” to describe the logarithmic decrement parameter in the MyotonPRO device is conceptually inaccurate. The logarithmic decrement quantifies the rate of energy dissipation in a damped oscillatory system—a phenomenon governed by both the restorative (elastic) and dissipative (viscous) components of material behaviour. As the review highlights, soft biological tissues exhibit viscoelastic properties, where part of the energy is stored elastically and part is dissipated as heat through internal friction and viscous flow. Therefore, referring to the logarithmic decrement as a measure of “elasticity” overlooks its dependence on damping and energy loss mechanisms intrinsic to viscoelastic systems. To align with current scientific understanding, the manufacturer’s terminology should be revised to reflect “viscoelasticity”, as this more accurately encompasses both the stiffness and damping characteristics that the logarithmic decrement represents.

Overall, the effects of TMR remain a subject of debate in the literature, likely due to subtle differences in study design. Our finding that TMR did not produce immediate biomechanical changes after a single intervention is consistent with some literature that also suggests the effects of rolling may have a delayed onset [24,41]. These findings collectively indicate that foam rolling does affect tissue stiffness, but its effects appear to be delayed rather than immediate. Therefore, although TMR is praised for its convenience and potential for frequent use, our results suggest that, at least in the single-intervention protocol of this study, the immediate biomechanical changes achieved by MMR may not be replicated by TMR techniques. This is because the applied force, direction, and depth may differ fundamentally and involve different mechanisms of action. A significant interaction effect between Time and Group for all three parameters (elasticity, stiffness, and tone) further emphasizes this difference, indicating a fundamental divergence in how the two groups responded to the intervention.

A key finding of this study is the significant difference in the percentage change across all three measured parameters between the MMR and TMR groups. The MMR group consistently showed a negative percentage change, indicating an immediate reduction in muscle stiffness, tone, and elasticity, while the TMR group showed negligible changes. This stark difference in outcomes can be explained by the fundamental biomechanical principles at play [43,44]. A therapist applies concentrated, high force, whereas a foam roller disperses weight over a broad area, failing to achieve the same depth or intensity. This targeted force creates a deeper mechanical effect, directly influencing collagen fibers and immediately reducing resistance. It also triggers stronger neural reflexes, such as decreased gamma motor neuron activity, which directly lowers muscle tone and stiffness [35]. The efficacy of MMR also extends beyond biomechanics, relying on the therapist-patient dynamic. A skilled therapist uses palpation to precisely locate and apply sustained force to specific trigger points, a nuance a foam roller cannot replicate. The therapeutic benefit is even influenced by the biological effects of touch, impacted by the physiological and psychological states of both parties [37]. These combined effects, unique to a therapist’s intervention, are likely crucial for the immediate changes observed in the MMR group.

Interestingly, our analysis also revealed a significant main effect of gender on stiffness and tone but no significant interaction with Time or Group. A study with collegiate soccer players (equal numbers of men and women) using low- or high-force foam rolling to relax the hamstrings found no significant difference in treatment effects based on force or gender [45]. This finding is consistent with prior research and suggests that there may be inherent differences in these muscle properties between males and females, independent of the intervention itself.

Despite providing valuable insights, this study has limitations. A major limitation of this study is the potential for performance and detection bias, which stems from the lack of blinding. Due to the nature of the interventions, both the participants and the principal investigator were aware of group assignments. The same operator who administered the treatments also performed the immediate post-intervention MyotonPRO measurements. While every effort was made to ensure objective measurements, it is acknowledged that the operator’s unconscious expectations could have influenced data collection. Future research should use independent, blinded outcome assessors to mitigate this risk. The sample size is relatively small, and the participants were all young, healthy physical therapy students, which may limit the generalizability of the findings to a broader, more diverse population (especially those with chronic conditions). Given that their unique characteristics differ from the general public or individuals with low back pain, the findings may not be applicable to these broader groups. However, the choice of a homogenous group was intentional for this initial study, which aimed to explore the biomechanical differences between two distinct interventions (MMR vs. TMR). This approach helped to control for potential confounding variables. Nonetheless, future research should include a larger, more diverse participant group, including individuals diagnosed with low back pain, to better understand the long-term effects of MMR and TMR. The absence of a significant immediate effect from a single TMR session in this study highlights a clear direction for future research. Subsequent investigations should focus on exploring the dose–response relationship of TMR by manipulating key intervention variables such as duration, frequency, and applied force. This would help determine whether a cumulative effect is necessary to achieve biomechanical changes in the erector spinae muscles.

## 5. Conclusions

This study indicates that MMR significantly and immediately reduces the stiffness and tone of the erector spinae muscle, a change not observed in the TMR group. This implies that while TMR offers a valuable option for at-home care, it may not be a direct substitute for the immediate biomechanical effects achieved by a trained therapist. Nevertheless, it is acknowledged that due to the highlighted limitations of the current study design, its generalizability to clinical practice will require further research validation.

## Figures and Tables

**Table 1:** Demographic and physical characteristics of the manual (MMR) and tool-assisted (TMR) groups.

	Group	
	MMR	TMR
Female		
Number	10	6
Age (years)	21.10 ± 2.60	21.00 ± 0.00
Weight (kg)	50.80 ± 5.49	53.67 ± 3.93
Height (m)	1.59 ± 0.06	1.58 ± 0.06
BMI	20.10 ± 1.64	21.53 ± 1.62
Male		
Number	5	9
Age (years)	20.40 ± 2.19	21.67 ± 1.58
Weight (kg)	70.60 ± 10.78	67.89 ± 9.61
Height (m)	1.75 ± 0.05	1.73 ± 0.06
BMI	22.95 ± 2.49	21.49 ± 2.43

Tested by independent *t*-test between two groups.

**Table 2:** Mixed-model ANOVA results between- and within-subject effects on muscle properties.

	Elasticity				Stiffness				Tone			
Source	df	F	*p*	η^2^	df	F	*p*	η^2^	df	F	*p*	η^2^
Between-subjects effects												
Gender	1	2.284	0.143	0.081	1	5.503	0.027 *	0.175	1	4.329	0.047 *	0.143
Group	1	0.322	0.575	0.012	1	0.266	0.611	0.010	1	2.420	0.132	0.085
Gender × Group	1	0.063	0.804	0.002	1	1.339	0.258	0.049	1	2.774	0.108	0.096
Error	26				26				26			
Within-subjects effects												
Time	1	3.058	0.092	0.105	1	18.772	<0.001 ^†^	0.419	1	2.575	0.121	0.090
Time × Gender	1	0.015	0.903	0.001	1	1.137	0.296	0.042	1	0.746	0.396	0.028
Time × Group	1	7.023	0.014 *	0.213	1	10.107	0.004 ^†^	0.280	1	12.643	0.001 ^†^	0.327
Time × Gender × Group	1	0.901	0.351	0.033	1	0.005	0.943	0.000	1	0.719	0.404	0.027
Error (Time)	26				26				26			

*: *p* < 0.05; ^†^: *p* < 0.0125.

**Table 3:** Comparison of Myoton parameters (elasticity, stiffness, and tone) within each group before and after intervention.

			Pre-Intervention	Post-Intervention	Percentage Change (%)	^a^ Comparison Between Pre and Post-Intervention		
Myoton	Group	n	Mean ± SD	Mean ± SD	Mean ± SD	*df*	T	*p* Value
Elasticity	MMR	15	1.046 ± 0.242	0.979 ± 0.222	−6.389 ± 8.514	14	2.747	0.016
(logarithmic decrement)	TMR	15	0.988 ± 0.200	1.006 ± 0.227	1.715 ± 7.402	14	−0.980	0.344
	*^b^ p* value		0.482	0.738	< 0.0125			
Stiffness	MMR	15	258.831 ± 34.495	233.847 ± 37.19	−9.813 ±5.769	14	6.589	<0.0125
(N/m)	TMR	15	267.650 ± 68.849	264.628 ± 65.44	−0.519 ± 7.089	14	0.652	0.525
	*^b^ p* value		0.661	0.127	<0.0125			
Tone	MMR	15	14.597 ± 0.691	14.092 ± 0.922	−3.489 ± 3.730	14	3.639	<0.0125
(Hz)	TMR	15	15.168 ± 1.678	15.340 ± 1.701	1.191 ± 2.411	14	−1.835	0.088
	*^b^ p* value		0.233	0.019	<0.0125			

^a^ tested by paired *t*-test within each group; *^b^* tested by independent *t*-test between two groups The significance level for post hoc tests was adjusted to *p* < 0.0125 using the Bonferroni correction.

## Data Availability

The data that support the findings of this study are available from the corresponding author upon request.

## Abbreviations

The following abbreviations are used in this manuscript:

BMI	Mass index
EMG	Electromyography
MMR	Manual myofascial release
RCT	Randomized controlled trial
TMR	Tool-assisted myofascial release
